# Predicting survival for patients with mesothelioma: development of the PLACE prognostic model

**DOI:** 10.1186/s12885-023-11180-y

**Published:** 2023-07-26

**Authors:** Yuan Zhang, Nan Li, Ran Li, Yumei Gu, Xiaofang Liu, Shu Zhang

**Affiliations:** 1 Department of Respiratory and Critical Care Medicine, Beijing Institute of Respiratory Medicine and Beijing Chao-Yang Hospital, Capital Medical University, 8 Gongtinan Rd, Chaoyang District, Beijing, 100020 China; 2 Present Address: Department of Pulmonary and Critical Care Medicine, Beijing Hospital, Institute of Geriatric Medicine, National Center of Gerontology, Chinese Academy of Medical Sciences, Beijing, 100005 China; 3 Department of Radiology, Beijing Chao-Yang Hospital, Capital Medical University, 8 Gongtinan Rd, Chaoyang District, Beijing, 100020 China; 4 Department of Respiratory and Critical Medicine, Beijing Tongren Hospital, Capital Medical University, Beijing, 100005 China; 5 Department of Pathology, Beijing Chao-Yang Hospital , Capital Medical University, 8 Gongtinan Rd, Chaoyang District, Beijing, 100020 China

**Keywords:** Malignant pleural mesothelioma, Prognosis, Prediction model, Calcium, Lymphocyte

## Abstract

**Introduction:**

The overall survival of patients with mesothelioma is poor and heterogeneous. At present, the prediction model for Chinese patients needs to be improved. We sought to investigate predictors of survival in malignant pleural mesothelioma and develop prognostic prediction models.

**Methods:**

This Two-center retrospective cohort study recruited patients with pathologically diagnosed mesothelioma at Beijing Chao-Yang Hospital and Beijing Tong-Ren Hospital. We developed a new prognostic prediction model based on COX multivariable analysis using data from patients who were recruited from June 1, 2010 to July 1, 2021 in Beijing Chao-Yang Hospital (n = 95, development cohort) and validated this model using data from patients recruited from July 18, 2014 to May 9, 2022 in Beijing Tong-Ren Hospital (n = 23, validation cohort). Receiver operating characteristic analysis was used to estimate model accuracy.

**Results:**

The parameters in this new model included PLT > 289.5(10^9/L) (1 point), Lymphocyte > 1.785(10^9/L) (-1point), Age > 73 years old (1 point), Calcium > 2.145(mmol/L) (-1point), Eastern Cooperative Oncology Group performance status (ECOG PS) > 2 (2 points). When the sum of scores < 0, it is recognized as a low-risk group; when the score is 0 ~ 3, it is recognized as a high-risk group. The survival rate of patients in the high-risk group was significantly lower than that in the low-risk group (hazard ratio [HR], 3.878; 95% confidence interval [CI], 2.226–6.755; P < 0.001). The validation group had similar results (HR,3.574; 95%CI,1.064–12.001; P = 0.039). Furthermore, the areas under the curve 6 months after diagnosis in the two cohorts were 0.900 (95% CI: 0.839–0.962) and 0.761 (95% CI: 0.568–0.954) for development and validation cohorts, respectively.

**Conclusion:**

We developed a simple, clinically relevant prognostic prediction model for PLACE by evaluating five variables routinely tested at the time of diagnosis. The predictive model can differentiate patients of Chinese ethnicity into different risk groups and further guide prognosis.

## Introduction

Substantial changes in the 2021 WHO Classification of Tumours of the Pleura and Pericardium since the 2015 WHO Classification. The most important two points about mesothelioma include that (1) localized and diffuse mesothelioma no longer include the term “malignant” as a prefix;(2) the three main histologic subtypes (i.e., epithelioid, biphasic, and sarcomatoid) remain the same but architectural patterns and cytologic and stromal features are more formally incorporated into the 2021 classification on the basis of their prognostic significance [1].

Previous studies have shown that mesothelioma is a primary pleural tumour associated with asbestos exposure with highly aggressive features [1–3]. The median survival time of untreated patients fluctuated from 8 to 14 months [4]. However, there is heterogeneity in patient survival among different populations [5, 6]. Mesothelioma is incurable, and it is clinically considered that the surgical effect is not good enough [7, 8]. Currently accepted chemotherapy regimens may improve survival by several months [9, 10]. Immunotherapy is also gradually becoming a prospect for mesothelioma treatment [11–13]. However, the treatment may have side effects such as vomiting and bone marrow transplantation, and at the same time, it will also increase the economic burden, and ultimately lead to a decrease in the quality of life of patients. It is difficult to make rational medical decisions when the exact survival period of some patients cannot be estimated [14]. Therefore, it is necessary to analyse the independent predictive factors related to the prognosis of patients with mesothelioma, and further establish a prognosis prediction model to predict the possible survival time of patients. However, this is still challenging.

Some prognostic inflammation indices like the neutrophil-to-lymphocyte ratio (NLR), the platelet-to-lymphocyte ratio (PLR), the lymphocyte-to-monocyte ratio (LMR) have been determined as independent prognostic factors [15–18]. At the same time, many prognostic prediction models that are inflammation-based have also been tested to determine the prognosis and guide multimodality treatment regimens [19–24].

For example, Fraser J. H. Brims establishes a predictive score based on whether the patient loses weight, histological type, Eastern Cooperative Oncology Group performance status (ECOG PS), and peripheral blood hemoglobin and albumin indicators to predict the 18-month prognosis of unselected mesothelioma patients [25]. Beow Y. Yeap developed a prognostic prediction model based on mesothelioma prognostic tests, molecular subtypes, tumor volume and NLR in order to assess the expected outcome of newly diagnosed patients [26]. However, those predictors or models have not been widely used and verified in clinic yet for various reasons.

Our research group has previously evaluated the predictive performance of LENT [27] and BRIMS scores [25] based on the clinicopathological information of Chinese patients, and concluded that although the above models can stratify the prognostic risk of mesothelioma patients, the predictive performance still needs to be improved [28].

It is recognized that China is a country that produces a lot of asbestos. The incidence rate of malignant mesothelioma may continue to increase in the next decade [29]. The standardized incidence rate in 2016 was 0.53/1 million [30]. However, there is currently no prognostic prediction model for Chinese mesothelioma patients. Secondly, the ability of the published foreign prediction models to stratify the prognosis of Chinese mesothelioma patients still needs to be improved. Therefore, it is very urgent and important to comprehensively consider clinical, pathological and laboratory indicators to develop a prognosis prediction model to guide clinical decision-making. Therefore, we collected the information of patients from two centers in China. On the one hand, we observed the characteristics of mesothelioma patients in China, and on the other, we established and validated a prognostic model for the Chinese mesothelioma population.

## Methods

### Study population

In this retrospective study, patients with mesothelioma in Beijing Chaoyang Hospital affiliated to Capital Medical University from June 1, 2010 to July 1, 2021 were screened as a development cohort. Patients recruited from July 18, 2014 to May 9, 2022 in Beijing Tong-Ren Hospital were enrolled in the validation cohort. For both the development and validation cohorts, patients who met the following inclusion criteria were enrolled in the retrospective observational study. The inclusion criteria were older than 18 years old and diagnosed with mesothelioma by pathological examination. Exclusion criteria included a history of other malignancies or lack of laboratory information. Patients who ultimately entered the study were determined according to the inclusion and exclusion criteria, and all enrolled patients signed written informed consent. This study was approved by the ethics committee of Beijing Chao-Yang Hospital, Capital Medical University. This study population in the development cohort had previously been reported [28].

### Data collection

Demographic characteristics, clinical characteristics, laboratory indicators at the time of diagnosis but not treatment and follow-up survival information of all enrolled patients were comprehensively collected from the electronic medical record system. Demographic indicators included gender and age, clinical characteristics included ECOG PS score, smoking history, asbestos exposure history, diagnosis method, histological subtype, and treatment method. Laboratory indicators mainly included three aspects. The first part included various blood cell indicators in complete blood cytology. The second part included liver function, kidney function, blood lipid, protein and other indicators in biochemistry. The third part included prothrombin time (PT), activated partial thromboplastin time (APTT) and other indicators in coagulation. For the development and validation cohorts, we collected all examination information in the electronic medical record system from diagnosis to December 31, 2021 and August 1, 2022 respectively, and then all patients were followed up by telephone to collect the examination and prognosis information of patients in other hospitals. Finally, the survival status of the patients was determined.

### Statistical analysis

Continuous variables that were not normally distributed were represented by the median and interquartile range (IQR). Normally distributed continuous variables were shown as mean ± standard deviation (SD). We calculated the optimal cut-off value for continuous variables based on the Receiver Operating Characteristic (ROC) curve, and transformed the continuous variable into a categorical variable according to the optimal cut-off value. Categorical variables were expressed as frequencies and percentages. Kaplan Meier was used to draw survival curves and log-rank test for differences between the two groups. Univariate and multivariate analyses were performed by Cox logistic regressions. Univariate survival analysis was applied to detect potential independent factors associated with prognosis, and variables with P-values < 0.05 were further included to a multivariate Cox proportional hazards model. We evaluate the points of each variable based on the magnitude of the regression coefficient. The sub term of each factor was the regression coefficient of the model divided by the minimum coefficient, and rounded to the nearest integer. Given the aim of the model was to provide prognostic information at diagnosis when this treatment data was not available. ROC curves were also used to evaluate the performance of predictive models. All statistical analyses were performed using SPSS software (version 25.0; IBM, Armonk, NY, USA). This study was reviewed by a professional epidemiologist.

## Results

### Patient characteristics

In the development cohort, between June 1, 2010 and July 1, 2021, a total of 101 patients were pathologically diagnosed as mesothelioma in Beijing Chaoyang Hospital. Six patients were excluded for the following reasons. One patient’s laboratory results were missing and two patients’ specific subtypes could not been identified because the biopsy tissues were too small. Three patients were diagnosed as desmoplastic mesothelioma. Eventually 95 patients were included in the development cohort. Of the 95 patients, 49(51.6%) were male and 46(48.4%) were female. The median age was 64.2 years. ECOG PS was performed on all patients, eight patients (8.4%) had an ECOG PS of 0, 68 (71.6%) patients had an ECOG PS score of 1, 12 (12.6%) patients had an ECOG PS score of 2, and 7 (7.4%) patients had an ECOG PS score of 3. Thirty-nine patients (41.1%) had a history of smoking, and 26 patients (27.4%) had a clear history of asbestos exposure. 3(2.1%), 15(15.8%), 68(71.6%), and 10(10.5%) patients were diagnosed by cell blocks from malignant pleural effusion, ultrasound-guided percutaneous biopsy, medical thoracoscopy, and Video-Assisted Thoracic Surgery (VATS), respectively. Eighty-one patients (85.2%) were epithelioid subtypes. Seven patients (7.4%) were sarcomatoid subtypes. Seven patients (7.4%) were biphasic subtypes. Eighty-eight patients (92.6%) received chemotherapy ± anti-angiogenesis therapy (Table 1).

**Table 1:** Baseline demographic and clinical characteristics of the study population in the development and validation cohorts

Characteristic	Development cohort, n = 95	Validation cohort, n = 23	P value
**Age (Mean ± SD)**	64.2 ± 9.6	61.7 ± 8.3	0.259
**Gender**			0.423
Male	49(51.6)	14(60.9)	
Female	46(48.4)	9(39.1)	
**ECOG PS**			< 0.001
0	8(8.4)	14(60.9)	
1	68(71.6)	9(39.1)	
2	12(12.6)	0(0)	
3	7(7.4)	0(0)	
**Smoke history**			0.349
Never	56(58.9)	16(69.6)	
Current and former	39(41.1)	7(30.4)	
**Asbestos exposure**			0.018
No	69 (72.6)	22(95.7)	
Yes	26(27.4)	1(4.3)	
**Diagnostic methods**			0.127
Cell blocks from malignant pleural effusion	3(2.1)	0(0)	
Ultrasound guided percutaneous biopsy	15(15.8)	1(4.3)	
Medical thoracoscopy	68(71.6)	21(91.4)	
Video-Assisted Thoracic Surgery	10(10.5)	1(4.3)	
**Histology**			0.117
Epithelioid	81(85.2)	17(73.9)	
Sarcomatoid	7(7.4)	5(21.7)	
Biphasic	7(7.4)	1(4.3)	
**Treatment**			< 0.001
Best supportive care	7(7.4)	10(43.5)	
Chemotherapy ± anti-angiogenesis therapy	88(92.6)	13(56.5)	
**ESR (mm/h)**	15.000(7.500,24.500)	43.390 ± 32.287	< 0.001
**Serum sodium(mmol/L)**	140.9 ± 3.1	139.8 ± 2.4	0.114
**Serum potassium(mmol/L)**	4.0 ± 0.4	4.1 ± 0.4	0.190
**Serum calcium(mmol/L)**	2.2(2.1,2.3)	2.2 ± 0.1	0.499
**WBC count(10^9/L)**	6.3(5.0,8.3)	7.4(5.2,8.8)	0.265
**Neutrophil(10^9/L)**	4.1(3.1,5.8)	4.5(3.3,6.2)	0.343
**Lymphocyte(10^9/L)**	1.5(1.2,1.9)	1.5(1.2,1.9)	0.200
**Monocyte(10^9/L)**	0.4(0.3,0.6)	0.6(0.4,0.7)	0.630
**Erythrocyte(10^9/L)**	4.5(4.1,4.8)	4.5 ± 0.4	0.596
**Haemoglobin (g/L)**	131.5 ± 17.4	132.6 ± 15.3	0.789
**Platelet(10^9/L)**	247.0(189.0,294.0)	263.0(210.0,417.0)	0.219
**Prealbumin(g/L)**	0.204(0.150,0.240)	0.192 ± 0.093	0.975
**Total protein(g/L)**	63.900(60.600,68.800)	66.065 ± 8.252	0.248
**AST(U/L)**	19.000(16.000,22.000)	18.087 ± 6.643	0.247
**ALT(U/L)**	16.000(14.000,22.000)	15.000(11.000,22.000)	0.624
**Creatine Kinase(U/L)**	55.000(40.000,70.000)	72.000(43.000,92.000)	0.224
**Triglycerides(mmol/L)**	1.290(0.900,1.880)	1.160(0.880,1.500)	0.418
**cholesterol(mmol/L)**	4.439 ± 1.114	4.700 ± 0.889	0.299
**HDL(mmol/L)**	1.060(0.900,1.250)	0.960(0.770,1.100)	0.191
**LDL(mmol/L)**	2.730 ± 0.861	2.886 ± 0.890	0.439
**glucose(mmol/L)**	4.990(4.550,6.030)	2.660(2.310,3.410)	0.349
**Creatinine(umol/L)**	60.700(51.100,72.700)	5.968 ± 2.037	0.787
**Uric acid(umol/L)**	301.000(239.570,361.000)	63.643 ± 11.337	0.747
**Urea Nitrogen(mmol/L)**	4.948 ± 1.502	4.760 ± 1.168	0.577
**Osmotic pressure(mOSM /L)**	283.000(279.000,286.000)	287.844 ± 10.297	0.060
**TT(s)**	18.600(17.100,19.400)	16.461 ± 1.512	< 0.001
**PT(s)**	11.800(10.900,12.400)	11.830 ± 0.863	0.964
**APTT(s)**	27.800(25.200,31.800)	27.835 ± 3.280	0.575
**Fibrinogen(mg/dl)**	3.849(3.053,4.596)	4.760 ± 2.088	0.849

Twenty-three patients were included in the validation cohort. Of the 23 patients, 14(60.9%) were male and 9(39.1%) were female. The median age was 61.7 years. ECOG PS was performed on all patients, fourteen patients (60.9%) had an ECOG PS of 0, 9 (39.1%) patients had an ECOG PS score of 1. Seven patients (30.4%) had a history of smoking. Only one patient (4.3%) had a clear history of asbestos exposure. Twenty-one patients were diagnosed by medical thoracoscopy, and the other two patients were diagnosed by ultrasound-guided percutaneous biopsy and VATS respectively. Seventeen patients (73.9%) were epithelioid subtypes. 5 patients (21.7%) were sarcomatoid subtypes.1 patient (4.3%) was biphasic subtype. Thirteen patients (56.5%) received chemotherapy ± anti-angiogenesis therapy (Table 1).

Available hematological indices of complete blood cytology, biochemistry, coagulation in these two cohorts were listed in Table 1. As indicated by the chi-square test and independent-sample T test, most baseline variables and laboratory examinations in the development cohort were similar to those in the validation cohort (Table 1).

### Development of the new PLACE prognostic score model

In the development cohort, during the 57 (28,100) months of follow-up, all patients’ median survival time was 24 (12, 52) months. 67 patients died during follow-up and 28 patients were still alive. Univariate analysis showed that age, ECOG PS, histology, erythrocyte sedimentation rate (ESR), serum calcium, lymphocyte, monocyte, hemoglobin, platelet, prealbumin, high density lipoprotein (HDL), PT, fibrinogen might have a significant correlation with the prognosis of mesothelioma (Table 2). Indicators with significant differences in univariate were included into the multivariate analysis. Finally, age (HR: 2.017; 95%CI: 1.114–3.652; P = 0.021), ECOG-PS (HR: 3.392; 95%CI: 1.288–8.937; P = 0.013), serum calcium (HR: 0.430; 95%CI: 0.253–0.732; P = 0.002), lymphocyte (HR: 0.446; 95%CI: 0.209–0.950; P = 0.036), PLT (HR: 1.935; 95%CI: 1.002–3.738; P = 0.049) were determined as an independent risk factor for prognosis in patients with mesothelioma (Table 3).

**Table 2:** Univariable Cox regression analyses for the prognosis of malignant pleural mesothelioma patients in the development cohort

Characteristic	HR	95%CI	P value
Age			
≤ 73	1		
> 73	2.229	1.249–3.979	0.007
**ECOG PS**			
0–2	1		
3	2.611	1.174–5.084	0.019
**Histology**			
Sarcomatoid	1		
Non-sarcomatoid	3.757	1.423–9.918	0.008
**ESR(mm/h)**			
≤ 31.500	1		
> 31.500	2.444	1.420–4.205	0.001
**Serum calcium(mmol/L)**			
≤ 2.145	1		
> 2.145	2.204	1.349–3.602	0.002
**Lymphocyte(10^9/L)**			
≤ 1.785	1		
> 1.785	1.797	1.029–3.139	0.039
**Monocyte(10^9/L)**			
≤ 0.535	1		
> 0.535	1.943	1.174–3.216	0.010
**Haemoglobin(g/L)**			
≤ 124.5	1		
> 124.5	0.598	0.363–0.985	0.043
**Platelet(10^9/L)**			
≤ 289.5	1		
> 289.5	1.708	1.006–2.899	0.047
**Prealbumin(g/L)**			
≤ 0.244	1		
> 0.244	0.217	0.093–0.507	< 0.001
**HDL(mmol/L)**			
≤ 1.215	1		
> 1.215	0.470	0.259–0.853	0.013
**PT(s)**			
≤ 12.750	1		
> 12.750	2.222	1.238–3.989	0.007
**Fibrinogen(mg/dl)**			
≤ 3.810	1		
> 3.810	2.763	1.651–4.622	< 0.001

**Table 3:** Multivariate Cox regression analyses for the prognosis of malignant pleural mesothelioma patients in the development cohort

Characteristic	β	HR^a^	95%CI^b^	P value
**Age > 73(years)**	0.702	2.017	1.114–3.652	0.021
**ECOG PS > 2**	1.221	3.392	1.288–8.937	0.013
**Calcium > 2.145(mmol/L)**	-0.843	0.430	0.253–0.732	0.002
**Lymphocyte > 1.785(10^9/L)**	-0.808	0.446	0.209–0.950	0.036
**PLT > 289.5(10^9/L)**	0.660	1.935	1.002–3.738	0.049

According to age, ECOG PS, serum calcium, lymphocyte, PLT indicators, a PLACE model was established to predict the prognosis of mesothelioma patients. Patients received − 1 point when the following conditions were met: Calcium > 2.145(mmol/L), Lymphocyte > 1.785(10^9/L). The patient gets 1 point when the following conditions were met: Age > 73(years), PLT > 289.5(10^9/L). The patient gets 2 points when the following conditions were met: ECOG PS > 2 (Table 4).

**Table 4:** New score for the prognosis among malignant pleural mesothelioma patients in the development cohort

Characteristic	Risk score
**Age > 73(years)**	1
**ECOG PS > 2**	2
**Calcium > 2.145(mmol/L)**	-1
**Lymphocyte > 1.785(10^9/L)**	-1
**PLT > 289.5(10^9/L)**	1

**Fig. 1:**
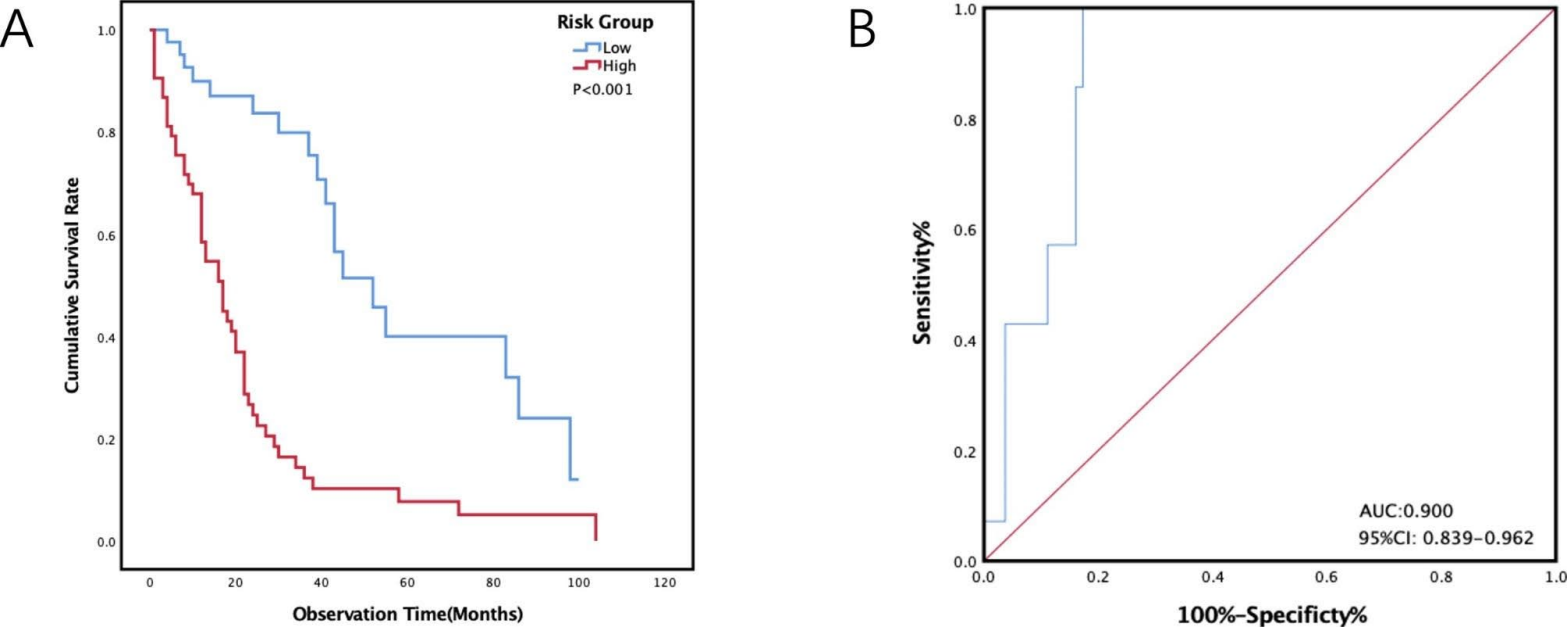
Kaplan-Meier curves (**A**) and receiver operating characteristic curves at 6 months (**B**) stratified by the new score group in the development cohort

In adding the scores for each item, patients were considered as the low risk when the sum of each score was between − 2 and − 1 points. When the sum is between 0 and 3, the patient was determined to be the high-risk group. Median survival in the low-risk group of 42 patients was 52(39, 86) months. The median survival time of the high-risk group consisting of 53 patients was 17 (8, 24) months (Table 5). The survival rate of the high-risk group was significantly lower than that of the low-risk group (HR: 3.878; 95% CI: 2.226–6.755; P < 0.001) (Table 5) (Fig. 1A). At 6 months after diagnosis, the area under curve (AUC) of the prognostic prediction model was 0.900 (95% CI 0.839–0.962) (Fig. 1B). Hosmer-Lemeshow showed χ2 = 5.601, P = 0.587.

**Table 5:** Median survival of different risk group patients in the development cohort

Variable	Score	Patients (%)	Survive time (Median, IQR)	HR	95%CI	P value
**Low Risk Group**	-2~-1	42(44.2)	52(39, 86)	1	-	-
**High Risk Group**	0~3	53(55.8)	17(8,24)	3.878	2.226–6.755	< 0.001

### Evaluation of the LENT and BRIMS score

**Fig. 2:**
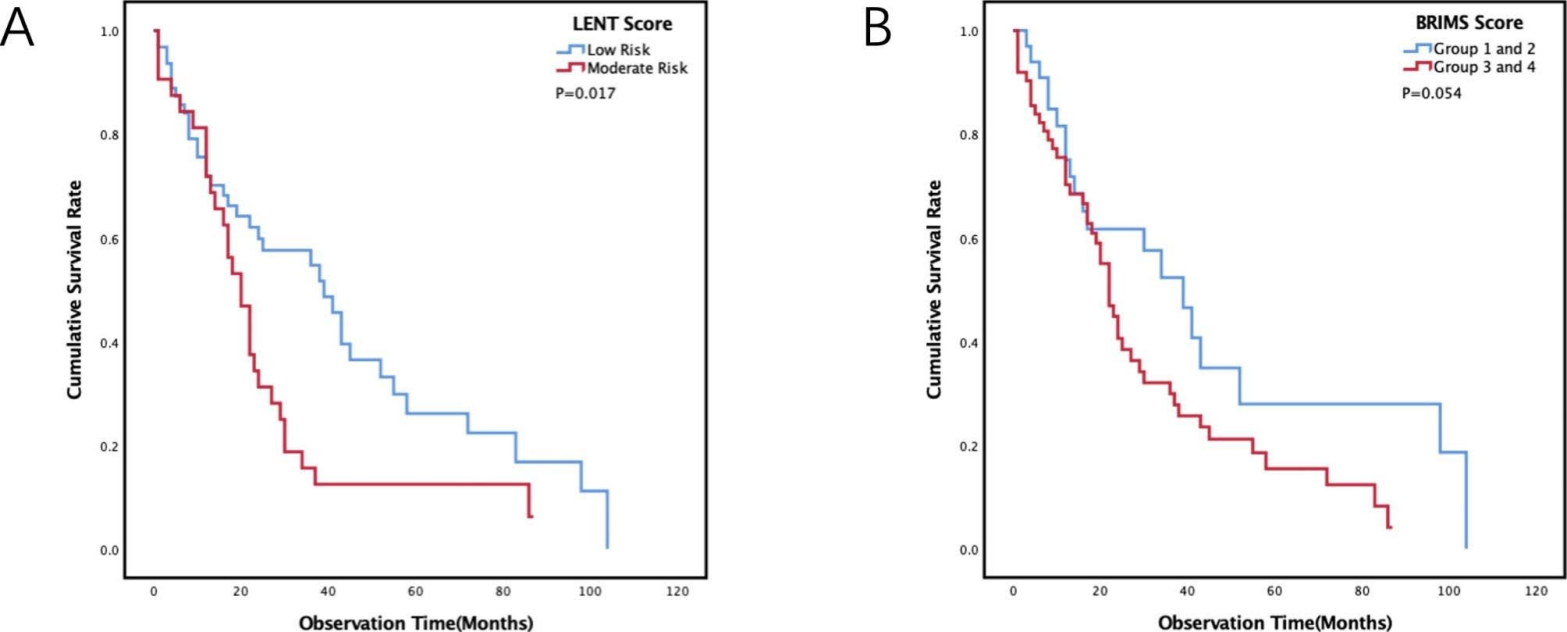
Kaplan-Meier curves stratified by the LENT score (**A**) and BRIMS score (**B**) in the development cohort

We classified the patients in the development cohort according to the LENT and BRIMS scoring standards and evaluated the prognostic stratification ability of these two scores in this cohort. The results showed that the LENT score could stratify the prognosis (P = 0.017) (Fig. 2A), but the BRIMS score could not effectively stratify the prognosis (P = 0.054) (Fig. 2B). However, both of these scores’ KM curves intersect in the early stage of diagnosis.

### Validation of the new PLACE prognostic score model

**Fig. 3:**
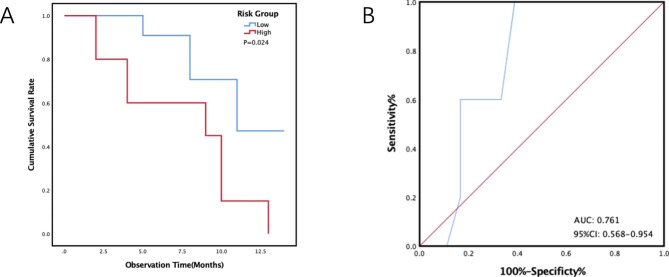
Kaplan-Meier curves (**A**) and receiver operating characteristic curves at 6 months (**B**) stratified by the new score group in the validation cohort

The new prognostic model was tested in a validation cohort of 23 patients, during the 10 (6,14) months of follow-up, all patients’ median survival time was 10 (8,13) months. 12 patients died during follow-up and 11 patients were still alive. Thirteen patients were determined to be the low-risk group. Median survival in the low-risk group was 11(8, -) months. There were ten patients in the high-risk group. The median survival time of the high-risk group was 9 (4, 10) months. The survival rate of the high-risk group was significantly lower than that of the low-risk group (HR: 3.574; 95% CI: 1.064–12.001; P = 0.039) (Table 6) (Fig. 3A). At 6 months after diagnosis, the AUC of the prognostic prediction model was 0.761 (95% CI 0.568–0.954) (Fig. 3B). Hosmer-Lemeshow showed χ2 = 4.286, P = 0.509.

**Table 6:** Median survival of different risk group patients in the validation cohort

Variable	Score	Patients (%)	Survive time (Median, IQR)	HR	95%CI	P value
**Low Risk Group**	-2~-1	13(56.5)	11(8,-)	1		
**High Risk Group**	0~3	10(43.5)	9(4,10)	3.574	1.064–12.001	0.039

## Discussion

This study mainly collected unselected Chinese patients diagnosed with mesothelioma over 10 years. By evaluating the laboratory parameters and clinical data routinely collected during diagnosis, a prognostic prediction model for evaluating age, ECOG PS, calcium, lymphocytes and platelets was established and validated. According to this model, mesothelioma patients were divided into two distinct prognostic risk groups. Prognosis of patients differs significantly between high and low risk groups.

### Characteristics of mesothelioma patients in China

In the development and validation cohort of this study, women accounted for 48.4% and 39.1% respectively which differed from most studies where the frequency was around 10-25% [31–33] in most studies. This study is a retrospective cohort study of two centres in China, and the sample size of patients involved is relatively small, so it is possible to be different from other studies in gender composition.

It is well known that there is a significant correlation between the occurrence of MPM and asbestos exposure [2, 3]. Most occupations exposed to asbestos require more intensive manual labour, such as the construction or shipbuilding industry. Therefore, men have more opportunities to obtain such jobs and are more likely to be exposed to asbestos. Only 22.9% of the patients in this study had a clear history of asbestos exposure. This also potentially suggests the reason for the low proportion of men in this study. However, in some studies, the proportion of women is as high as 40 − 50% [34, 35]. This suggests that the gender proportion of MPM varies in different regions. In the future, it is still necessary to further analyse and summarize the big data covering all regions of the world.

Interestingly, previous studies have shown that the prognosis of women with mesothelioma is generally better than that of men [36]. There are two possible reasons: on the one hand, the high estrogen receptor-beta expression on mesothelioma tumours has been shown to have better survival [37]. The expression of estrogen and estrogen receptor beta in female patients was significantly higher than that in male patients. On the other hand, women are generally more sensitive, so they will visit doctors earlier than men for diagnosis and treatment [38]. This also explained why the overall survival of patients in this study was better.

Because mesothelioma is a relatively rare tumour with no specific symptoms, and is usually initially diagnosed as an advanced stage, and most of the patients are elderly, the above factors foreshadow a poor prognosis. However, the average age of patients at the time of diagnosis in this study is younger than that in other studies [39, 40]. Analysing the reasons, we think that with the continuous improvement of the public’s awareness of health examination, routine physical examination is widely used in China. Therefore, more and more young people are diagnosed at the early stage of the disease, which also provides opportunities for follow-up treatment.

We also consulted some relevant literature on race and mesothelioma. The results showed that race may be significantly related to the incidence rate and survival rate of MPM. Specifically, the incidence rate of mesothelioma in white patients is higher than that in black patients and patients of other races [41]. Whites have been shown to be independently associated with poor overall survival [42]. This may be because black patients are more likely to be diagnosed in the later stage of the disease and are less likely to receive surgical treatment [43] due to limited economic conditions, higher education, etc.

In addition, the median survival time of patients in this study was significantly longer than that in other studies. We analyze the following reasons: Firstly, as we said above, female patients account for a large proportion in this study, and the prognosis of female patients is good. Secondly, previous studies have shown that the prognosis of patients with epithelioid mesothelioma was better than that of sarcomatoid mesothelioma [44]. Most of the patients in this study had epithelioid mesothelioma. Thirdly, most patients have young age, good ECOG PS and received chemotherapy and/or anti angiogenic therapy. The fourth point is that due to the rarity of mesothelioma patients, the time span of this study was very long, and some patients failed to have a general examination. The clinical tumor stage of 44 patients can be evaluated systemically. The results showed that all 44 patients were in stage I - III.

### Necessity of establishing prognostic prediction models

Mesothelioma is a rare but aggressive tumour of incompletely understood pathogenesis. In addition to asbestos exposure, it is very heterogeneous on a molecular level. Worldwide the incidence and mortality rates vary greatly [45, 46]. The management of mesothelioma remains complex. Despite the current treatment paradigm of multimodality therapy including chemotherapy, surgery, and radiation therapy, there was no breakthrough in treatment, it still has a poor prognosis.

In the past few decades, clinicians have conducted in-depth research on many indicators from different perspectives such as basic epidemiological variables, laboratory examination results, imaging features and pathological characteristics and established many different predictive models [39, 47, 48]. However, there is currently no specific prognosis prediction model for mesothelioma patients in China. Secondly, the ability of published Western predictive models to stratify the prognosis of Chinese patients with mesothelioma is very limited. Therefore, the necessity and importance of the prognostic prediction model developed in this study specifically for Chinese patients with mesothelioma is very significant.

The characteristic of this model was that it was a prognosis prediction model specifically targeting the comprehensive clinical characteristics and laboratory indicators of Chinese mesothelioma patients. The advantage was that the clinical and laboratory indicators in the model are daily monitoring content, and there was no need to add additional physical and economic burden to the patient. Moreover, the discrimination and calibration abilities of this model were relatively good. The disadvantage was that the included pathological and imaging features were not comprehensive. The limitation was that the sample size of the model establishment and validation cohort was small, and there were similarity in the population, which did not target all patients with mesothelioma. The potential problem in the use process lies in the low universality, and the ability to predict the prognosis of patients with another central mesothelioma remains to be further explored.

### Comparison with the LENT and BRIMS score

As we have described before [28], Although LENT could effectively stratify the patients with mesothelioma in China, BRIMS was difficult to effectively stratify the prognosis. However, the KM curves of both of these two scores crossed at the early stage of diagnosis. After 18 months of follow-up, the two risk groups began to separate. The reason might be related to the composition of special patient population. Therefore, the ability of these two foreign scores to evaluate the prognosis of mesothelioma in China was relatively limited, and its validation still needed to be further confirmed by large sample studies.

### Age and prognosis

Our multivariate analysis showed that the prognosis of elderly patients with mesothelioma was worse than that of young patients. This was consistent with the results of many previous studies [35, 47], which was easy to understand. Elderly patients generally had more basic diseases, poor pulmonary function, and limited access to treatment. However, the age factor was still a controversial topic. Because some researchers had reached the opposite conclusion [49, 50] who believed that the tumor invasiveness of elderly patients might also be low.

### ECOG PS and prognosis

Previous studies had shown that ECOG PS was a subjective assessment, as it is mainly based on activities of daily living, depending on the information provided by patients and caregivers [51]. It was usually the cornerstone of the prognosis of advanced cancer, and poor ECOG PS was an independent risk factor for the prognosis of solid tumours [52]. In this study, ECOG PS was significantly related to prognosis, so we included ECOG PS in the new prediction model. Most patients had good ECOG PS scores. Only some patients could not live normally due to a large amount of pleural effusion or basic diseases. The prognosis of such patients was usually poor.

### Calcium and prognosis

This study was the first clinical study to study the effect of calcium in peripheral blood on the prognosis of mesothelioma patients. This study suggested that low serum calcium levels might be significantly associated with poor prognosis in mesothelioma patients. Previous studies had shown that low serum calcium levels might lead to poor prognosis in patients with esophageal cancer [53], gastric cancer [54] and nasopharyngeal carcinoma [55]. However, some studies have reached the opposite conclusion [56, 57].It was well known that the low electrolyte concentration in blood tests was usually considered as an indicator of the overall poor condition of cancer patients. The low oral intake of tumour patients, large consumption of tumour inconvenient movement of patients might lead to a decrease in serum calcium levels. In addition, the causes might also include the occurrence of bone metastasis, use of chemotherapy drugs [58], tumour lysis syndrome [59]. In addition, tumour induced inflammation might change endothelial/vascular renal function, and might also lead to decreased calcium reabsorption [54]. It was reported that calcium homeostasis was involved in the regulation of cancer related biological processes including cell proliferation, tumorigenesis, migration and invasion, angiogenesis [60, 61]. Ultimately, the incidence of death increased. The above results indicated that calcium played an important role in the occurrence and development of cancer [60, 61]. However, only a few basic studies have shown that Cav3.2 [62] and KCa1.1 [63] are specifically expressed in mesothelioma tissue samples. Therefore, the specific correlation and mechanism between calcium and prognosis of mesothelioma patients still need to be further explored.

### Platelet and prognosis

The high level of platelet count was shown to be a bad influence factor on survival in this study, which has also been proved in our study corroborating with other studies [64–68]. The reason may be that tumor cells release a series of cytokines, stimulate megakaryocytes and lead to an increase in platelet levels [69]. In turn, platelets can protect cancer cells from the influence of high shear force, and can also form adhesion bridges between tumor cells and capillaries, thus promoting the growth, invasion and metastasis of tumors [70, 71]. Finally, it leads to poor prognosis of patients.

### Lymphocyte and prognosis

At present, the pathogenesis of mesothelioma has not been fully elucidated, since asbestos exposure is closely related to the occurrence of mesothelioma [72], it is speculated that the inflammatory response may promote tumorigenesis. Cellular and soluble components of the tumor microenvironment play critical roles in cancer development and prognosis [73]. Lymphocytes are an important part of the host immune system. They can activate potent anti-tumor cellular immune responses and inhibit micro-metastases. Elevated lymphocyte counts were significantly associated with clinical benefits from chemotherapy and immunotherapy in patients with mesothelioma [74–76].

This study has some limitations. First, selection bias is unavoidable due to the nature of retrospective studies with small sample sizes. The diagnosis time span of patients is large, and the development of treatment and technology may affect the outcome of the disease to a certain extent. Secondly, our study lacks relevant genetic molecular variables and imaging variables. Thirdly, the number of people in the validation cohort was small and the follow-up time was short, which might affect the effectiveness of validation. Fourthly, due to the large time span of patients included, most patients could not be given standard general examination in the early years, so most patients could not be determined accurate clinical stages. Fifth, our cohort was too restrictive in localized at only two hospital centres. Furthermore, the time frame for the validation cohort was not optimally overlapped with that of the development cohort, which leaves room for bias. In the future, we will actively seek multi-centre cooperation around the world, and simultaneously carry out prospective research in order to solve the problem mentioned above.

## Conclusion

This study has developed and verified a model specifically targeting pleural mesothelioma that is suitable for the regional characteristics of China—PLACE by evaluating the results of evaluating factors of age, ECOG PS, calcium, lymphocyte and PLT. Although this was established in a small cohort at a single facility, the above indicators are common clinical laboratory tests which are easy to obtain, highly repeatable and have uniform testing standards in different medical institutions. The new model can classify patients into two risk subgroups. There were significant differences in the prognosis of patients in different risk groups. According to this model, clinicians could try to identify high-risk patients and choose more appropriate treatment options. However, the proposed model can only be applied to patients of Chinese ethnicity now and not currently generalizable to the overall global population. In addition, although patients from two hospitals were included in this study, the overall sample size was still small. Moreover, there are deviations between the development and validation cohorts. Therefore, the stratification ability of the new model should be further validated in the future prospective multi-centre and large sample studies worldwide.

## Data Availability

The datasets used and/or analysed during the current study are available from the corresponding author on reasonable request.

## Abbreviations

HR: Hazard radio
CI: Confidence interval
NLR: Neutrophil-to-lymphocyte ratio
PLR: Platelet-to-lymphocyte ratio
LMR: Lymphocyte-to-monocyte ratio
ECOG PS: Eastern Cooperative Oncology Group Performance Status
PT: Prothrombin time
APTT: Activated partial thromboplastin time
IQR: Interquartile range
SD: Standard deviation
ROC: Receiver Operating Characteristic
ESR: Erythrocyte sedimentation rate
AST: Aspartate Transaminase
HDL: High density lipoprotein
WBC: White blood cell
ALT: Alanine transaminase
LDL: Low density lipoprotein
AUC: Area under curve
TT: Thrombin time
